# Draft genome sequence of *Dyadobacter tibetensis* type strain (Y620-1) isolated from glacial ice

**DOI:** 10.4056/sigs.5649756

**Published:** 2014-03-15

**Authors:** Yongqin Liu, Anyi Hu, Liang Shen, Tandong Yao, Nianzhi Jiao, Ninglian Wang, Baiqing Xu

**Affiliations:** 1 Key Laboratory of Tibetan Environmental Changes and Land Surface Processes, Institute of Tibetan Plateau Research, Chinese Academy of Sciences, Beijing, China; 2 Key Laboratory of Urban Environment and Health, Institute of Urban Environment, Chinese Academy of Sciences, Xiamen, China; 3 State Key Laboratory of Marine Environmental Science, Xiamen University, Xiamen, China; 4 Cold and Arid Regions Environmental and Engineering Research Institute, Chinese Academy of Sciences, Lanzhou, China

**Keywords:** *Dyadobacter tibetensis*, draft genome, psychrotolerant bacterium, glacial ice

## Abstract

*Dyadobacter tibetensis* Y620-1 is the type strain of the species *Dyadobacter tibetensis*, isolated from ice at a depth of 59 m from a high altitude glacier in China (5670 m above sea level). It is psychrotolerant with growth temperature ranges of 4 to 35°C. Here we describe the features of this organism, together with the draft genome sequence and annotation. The 5,313,963 bp long genome contains 4,828 protein-coding genes and 39 RNA genes. To the best of our knowledge, this is the first *Dyadobacter* strain that was isolated from glacial ice. This study provides genetic information of this organism to identify the genes linked to its specific mechanisms for adaption to extreme glacial environment.

## Introduction

Strain Y620-1 (=JCM 18589= CGMCC 1.12215T) is the type strain of the species *Dyadobacter tibetensis* [1]. The genus *Dyadobacter* currently has 12 species after it was first proposed by Chelius and Triplett on 2000, and the type species is *D. fermentans* [2]. Those species isolated from diverse environment, i.e. glacial ice, soil from the Arctic, Colorado Plateau, farm and a ginseng field, desert sand, freshwater and sea water, and plant material [1-12]. So far, however, the genome sequences have been determined for only three *Dyadobacter* strains (*D. alkalitolerans* DSM 23607 (GCA_000428845), *D. fermentans* DSM 18053 (GCA_000023125), *D. beijingensis* DSM 21582 (GCA_000382205)), and only the complete genome sequence of *D. fermentans* DSM 18053 has been published [13].

*D. tibetensis* strain Y620-1 was isolated from 59 m depth section of an 122 m ice core drilled from Yuzhufeng Glacier at 5670 m above sea level, Tibetan Plateau, China [1]. Glacier ice is an extreme environment with low temperature and nutrients, but high UV radiation, and is a huge reservoir of extremophilic microorganism that have accumulated for hundreds of years [14]. Diverse isolates were recovered from glacial ice, but the genomes of bacteria in the extreme environment were limited [15,16]. Here, we present the genome sequence of psychrotolerant *D. tibetensis* strain Y620-1 isolated from ice core. This is the first genome sequence of a bacterial isolated from a deep high altitude glacier ice.

## Classification and features

The phylogenetic position of genus *Dyadobacter* is in the *Cytophagaceae*, a very diverse family within the order *Sphingobacteriales*, the phylum *Bacteroidetes* [13]. Closest related genera are *Persicitalea* and *Runella* [13]. *D. tibetensis* strain Y620-1 represents a novel species of the genus *Dyadobacter* based on reported genotypic and phenotypic feature [1].

**Figure 1:**
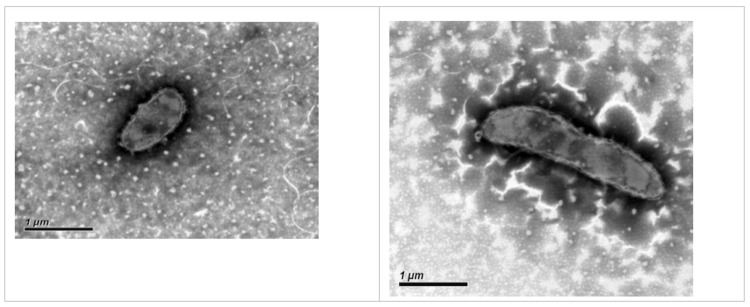
Transmission electron micrograph of *Dyadobacter tibetensis* (T) Y620-1

**Figure 2:**
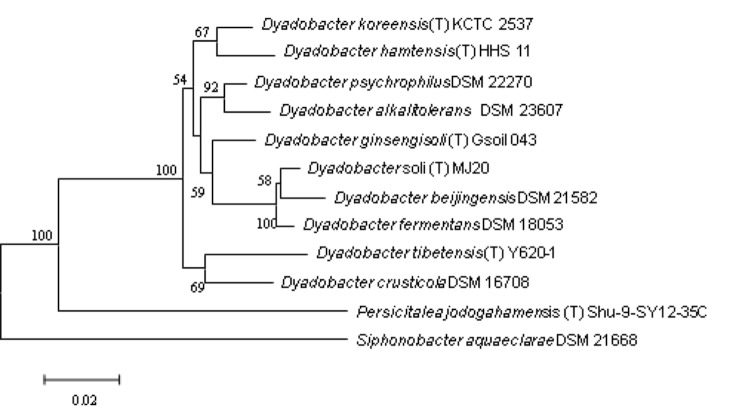
Phylogenetic tree highlighting the position of *Dyadobacter tibetensis* (T) Y620-1 relative to other type strains within the genus *Dyadobacter*. Type strains *D. alkalitolerans* DSM 23607, *D. beijingensis* DSM 21582 and *D. fermentans* DSM 18053 are having fully sequenced genomes with assigned GenBank Assembly ID GCA_000428845.1, GCF_000382205.1 and GCA_000023125.1. The type strains and their corresponding GenBank accession numbers for 16S rRNA genes are: *D. ginsengisoli* Gsoil 043(T), AB245369*; D. crusticola,* DSM 16708, AJ821885*; D. alkalitolerans* DSM 23607, EU360597; *D. psychrophilus* DSM 22270, GQ131577; *D. koreensis* KCTC 12534(T), EF017660; *D. soli* MJ20(T), GQ241324; *D. fermentans* DSM 18053, CP001619; *D. hamtensis*HHS 11(T), AJ619978; *D. beijingensis* DSM 21582, DQ335125; *Persicitalea jodogahamensis* (T) Shu-9-SY12-35C, AB272165. The tree uses sequences aligned by the RDP aligner, and uses the Jukes-Cantor corrected distance model to construct a distance matrix based on alignment model positions without the use of alignment inserts, and uses a minimum comparable position of 200. The tree was built with RDP Tree Builder, which uses Weighbor [21] with an alphabet size of 4 and length size of 1000. Building of the tree also involves a bootstrapping process repeated 100 times to generate a majority consensus tree [22]. *Siphonobacter aquaeclarae* DSM 21668, FJ177421, was used as an out group.

Strain Y620-1 was isolated at 4°C incubation from ice core melt water cultivated on R2A medium [17]. It was Gram-stain-negative, non-motile and rod-shaped with 1 μm to 2 μm length [1] (Figure 1). Colonies are yellow, round, smooth, convex and opaque on R2A after incubation at 30°C for 2–3 days. Growth occurs at 4-35°C on R2A, with an optimum at 30°C. It produces a flexirubin-like pigment, the same as the other species in the genus. The major fatty acids are summed feature 3 (C_16: 1ω7c_ and/or iso-C_15: 0,2-OH_), iso-C_15: 0_, C_16: 1ω5c_ and iso-C_17: 0 3-OH_. The predominant polar lipid is phosphatidylethanolamine [1].The important characteristics of the strain based on literature descriptions are summarized in Table 1. The strain exhibited a 16S rRNA gene sequence similarity with other members of the genus *Dyadobacter* ranging from 95.1% with *D. ginsengisoli* Gsoil 043 to 93.6% with *D. beijingensis* A54 (Figure 2).

**Table 1:** Classification and general features of *Dyadobacter tibetensis* (T) Y620-1 according to the MIGS recommendations

**MIGS ID**	**Property**	**Term**	**Evidence code**^a^
	Current classification	Domain *Bacteria* Phylum *Bacteroidetes* Class *Sphingobacteria* Order *Sphingobacteriales* Family *Cytophagaceae* Genus *Dyadobacter* Species *Dyadobacter tibetensis* Type strain Y620-1	TAS [18] TAS [18] TAS [18] TAS [18] TAS [19] TAS [2] TAS [1]
	Gram stain	Negative	TAS [1]
	Cell shape	Rod	TAS [1]
	Motility	Nonmotile	TAS [1]
	Sporulation	Non-sporulating	TAS [1]
	Temperature range	4-35°C	TAS [1]
	Optimum temperature	30°C	TAS [1]
	Carbon source	glucose, arabinose, mannitol mannose	TAS [1]
	Energy source	Not reported	NAS
MIGS-6	Habitat	59 m depth section of 122 m ice core	TAS [1]
MIGS-6.3	Salinity	0 -5% (NaCl m/v)	TAS [1]
MIGS-22	Oxygen	Aerobic	TAS [1]
MIGS-15	Biotic relationship	free-living	TAS [1]
MIGS-14	Pathogenicity	Not reported	NAS
MIGS-4	Geographic location	China	TAS [1]
MIGS-5	Sample collection time	2010	IDA
MIGS-4.1	Latitude	94º 14.77′ E	TAS [1]
MIGS-4.2	Longitude	35º 39.64′ N	TAS [1]
MIGS-4.3	Depth	59 m	TAS [1]
MIGS-4.4	Altitude	5670 m above sea level	IDA

a) Evidence codes - IDA: Inferred from Direct Assay; TAS: Traceable Author Statement (i.e., a direct report exists in the literature); NAS: Non-traceable Author Statement (i.e., not directly observed for the living, isolated sample, but based on a generally accepted property for the species, or anecdotal evidence). These evidence codes are from the Gene Ontology project [20].

**Table:** 

	

The utilization of carbon compounds by strain Y620-1 was determined using Generation-III microplates on an OmniLog phenotyping device (BIOLOG Inc., Hayward, CA, USA). The microplates were inoculated at 30°C with a cell suspension at a cell density of 95-96% turbidity and dye IF-A. Strain Y620-1 assimilates dextrin, D-maltose, D-trehalose, D-cellobiose, gentiobiose, D-melibiose, D-salicin, N-Acetyl-D-glucosamine, D-mannose, Glycyl-L-proline, L-alanine, L-histidine, L-serine, methyl, pyruvate, L-lactic acid, citric acid, α-Keto-glutaric acid, L-malic acid, propionic acid and acetic acid, but not stachyose, D-raffinose,N-Acetyl-β-dmannosamine, N-Acetyl-dgalactosamine, N-Acetyl-neuraminic acid, D-galactose, 3-methyl glucose, L-fucose, L-rhamnose, inosine, D-sorbitol, D-mannitol, D-arabitol, myo-Inositol, glycerol, D-glucose-6-PO4, D-aspartic acid, gelatin, L-arginine, L-pyroglutamic acid, pectin, L-galactonic acid lactone, D-gluconic acid, glucuronamide, mucic acid, quinic acid, D-saccharic acid, β-hydroxy-phenylacetic acid, D-lactic acid methyl ester, D-malic acid, γ-amino-butryric acid, β-hydroxy-D,L-butyric Acid and formic acid. It is sensitive to minocycline, potassium tellurite, nalidixic acid, lithium chloride, fusidic acid, D-serine and sodium bromate, but not troleandomycin, rifamycin, lincomycin, guanidine HCl, niaproof 4, vancomycin, tetrazolium violet, tetrazolium blue, aztreonam and sodium butyrate.

## Genome sequencing information

### Genome project history

The organism was selected for sequencing on the basis of it from extreme deep ice core from high altitude glacier. The shotgun genome sequencing project was completed in December 2012 and has been deposited at DDBJ/EMBL/GenBank under the accession number AZQN00000000. The version described here is the first version, AZQN01000000. The genome sequencing was carried out in Shanghai Majorbio Bio-pharm Technology Co., Ltd (Shanghai, China). A summary of the project information is shown in Table 2.

**Table 2:** Project information

**MIGS ID**	**Property**	**Term**
MIGS-31	Finishing quality	High-quality draft
MIGS-28	Libraries used	Two illumina paired-end libraries (170 bp and 800 bp insert size)
MIGS-29	Sequencing platforms	Illumina GAIIx
MIGS-31.2	Fold coverage	384 ×
MIGS-30	Assemblers	SOAPdenovo v1.05
MIGS-32	Gene calling method	Glimmer3, RAST
	Genbank ID	AZQN00000000
	Genbank Date of Release	January 31, 2014
	NCBI project ID	PRJNA230913
MIGS-13	Source material identifier	CGMCC 1.12215
	Project relevance	Glacial microbial ecology

### Growth conditions and DNA isolation

Cells of strain Y620-1 were harvested from R2A broth following 2 days incubation at 30 °C with shaking at 180 rpm. The genomic DNA of the strain was extracted according to the method previously described by Marmur et al. [23]. Extraction was started with 100 ml of 48 h culture, centrifuged at 4 °C and 10, 000 rpm for 15 min. Then, cells were washed three times with 5 ml sterile water. The washed cells were resuspended in 1,128 μL Tris-HCl buffer (10 mM) containing 1 mM EDTA (pH 8.0) and 20 μg lysozyme and incubated at 37 °C for 2 h. followed by adding of 6 μL proteinase K (20 mg/mL), 4 μL DNase-free RNase (10 mg/mL), 100 μL SDS (20% w/v) and the cell suspension was incubated at 55 °C for 3 h. The cell lysate was extracted twice with phenol/chloroform/isoamyl alcohol (25:24:1) and once with chloroform/isoamyl alcohol (24:1), and the aqueous layer was separated after centrifugation at 12,000 rpm for 15 min. The DNA was precipitated with 1 volumes of frozen anhydrous ethanol. The purity of genomic DNA was assessed by NanoDrop (2000c, Thermo) with OD 260:280 ratio of 1.8-2. The DNA was stock in TE (pH 8.0) for genome sequencing.

### Genome sequencing and assembly

The genome of strain Y620-1 was sequenced using an Illumina GAIIx instrument with two paired-end libraries (170 bp and 800 bp insert size). The raw sequencing data was processed to discard reads containing adaptor sequences, a high rate of ambiguity, and removing the sequence reads which were of low quality. A total of 2,041 Mb high-quality of Illumina data were obtained, providing approximately 384–fold coverage. The high-quality reads were assembled in silico using SOAPdenovo v1.05, resulting in 33 contigs (> 200 bp) with an N50 length of 797,100 bp.

### Genome annotation

The coding sequences (CDS) were predicted using Glimmer 3.02 [24], while tRNAscan-SE [25] and RNAmmer [26] were used to identify tRNA and rRNA, respectively. The genome sequence was also uploaded into the Rapid Annotation using Subsystem Technology (RAST) system [27] to check the annotated sequences. The functions of predicted protein-coding genes were then annotated through comparisons with the databases of NCBI-NR [28], COG [29], and KEGG [30]. The program TMHMM [31] and SignalP [32] were used to identify putative transmembrane helices and signal peptides.

## Genome properties

The Y620-1 draft genome sequence has a total of 5,313,963 bp with an average GC content of 43.44%. There are 4,867 predicted genes, of which 4,828 are protein-coding genes, and 39 are RNA genes. A total of 2,844 genes (58.91%) are assigned a putative function. The remaining genes were annotated as either hypothetical proteins or proteins of unknown functions. Using COG functional assignment, 70.55% of protein coding genes could be classified into 20 COG categories. The properties and the statistics of the genome are summarized in Tables 3 and 4. According to the subsystem-based annotation generated by RAST, ~ 32% protein-coding genes of strain Y620-1 could be assigned to 358 metabolic subsystems. The most abundant of the subsystems are related to carbohydrates (n=323, 7.2% of total protein-coding genes), following by amino acids and derivatives (n=272, 6.0%), cofactors, vitamins, prosthetic groups, pigments (n=184, 4.1%), protein metabolism (n=143, 3.2%), membrane transport (132, 2.9%) and respiration (131, 2.9%).

**Table 3:** Nucleotide content and gene count levels of the genome

**Attribute**	Value	% of total^a^
Genome size (bp)	5,313,963	100
DNA coding region (bp)	4,680,447	88.08
DNA G+C content (bp)	2,308,448	43.44
Total genes	4,867	100
RNA genes	39	0.80
Protein-coding genes	4,828	82.68
Genes with function prediction	2,844	58.91
Genes assigned to COGs	2,208	70.55
Genes assigned to Pfam domains	3299	68.33
Genes assigned to TIGRfam domains	2161	44.76
Genes with signal peptides	372	7.71
Genes with transmembrane helices	628	13.01
CRISPR repeats	1	

a) The total is based on either the size of the genome in base pairs or the total number of protein coding genes in the annotated genome.

**Table 4:** Number of genes associated with the 25 general COG functional categories

**Code**	**Value**	**%age**^a^	**Description**
J	149	3.09	Translation
A	0	-	RNA processing and modification
K	261	5.14	Transcription
L	130	2.69	Replication, recombination and repair
B	0	-	Chromatin structure and dynamics
D	22	0.46	Cell cycle control, mitosis and meiosis
Y	0	-	Nuclear structure
V	61	1.26	Defense mechanisms
T	217	4.49	Signal transduction mechanisms
M	288	5.97	Cell wall/membrane biogenesis
N	12	0.25	Cell motility
Z	1	0.02	Cytoskeleton
W	0	-	Extracellular structures
U	52	1.08	Intracellular trafficking and secretion
O	109	2.26	Posttranslational modification, protein turnover, chaperones
C	201	4.16	Energy production and conversion
G	269	5.57	Carbohydrate transport and metabolism
E	233	4.83	Amino acid transport and metabolism
F	73	1.51	Nucleotide transport and metabolism
H	182	3.77	Coenzyme transport and metabolism
I	132	2.73	Lipid transport and metabolism
P	259	5.36	Inorganic ion transport and metabolism
Q	84	1.74	Secondary metabolites biosynthesis, transport and catabolism
R	410	8.49	General function prediction only
S	261	5.41	Function unknown
-	1422	29.45	Not in COGs

a) The total is based on the total number of protein coding genes in the annotated genome.

## Discussion

Although there were 12 species assigned to *Dyadobacter*, only three have a completed genome sequence and are *D. alkalitolerans* DSM 23607, *D. fermentans* DSM 18053, and *D. beijingensis* DSM 21582. Strain Y620-1 has the smallest genome of *D. alkalitolerans* DSM 23607, *D. fermentans* DSM 18053 and *D. beijingensis* DSM 21582 (6.29 Mbp, 6.97 Mbp and 7.37 Mbp, respectively). The GC content of strain Y620-1 is comparable to that of *D. alkalitolerans* DSM 23607 (45.66%), but lower than those of *D. fermentans* DSM 18053 (51.54%) and *D. beijingensis* DSM 21582 (52.09%). In order to estimate the similarity among the sequenced *Dyadobacter* strains, an average nucleotide identity (ANI) and Genome-to-Genome Distance Calculator (GGDC) were calculated using the software JSpecies v1.2 [33] and GGDC v2.0 [34], respectively. Table 5 shows the results of ANI and GGDC.

**Table 5:** Pairwise comparisons between four *Dyadobacter* strains using the ANI and GGDC.

		**ANI**		**GGDC**		
		**ANIb (%)**	**ANIm (%)**	**HSP / total length (%)**	**Identities / HSP length (%)**	**Identities / total length (%)**
*D. tibetensis* Y620-1	*D. alkalitolerans DSM 23607*	68.04	83.14	13	18	13.3
*D. tibetensis* Y620-1	*D. fermentans DSM 18053*	67.86	83.88	13	18.1	13.4
*D. tibetensis* Y620-1	*D. beijingensis DSM 21582*	67.7	83.46	13	17.4	13.3
*D. alkalitolerans DSM 23607*	*D. fermentans DSM 18053*	72.89	82.23	16.5	18.2	16.3
*D. alkalitolerans DSM 23607*	*D. beijingensis DSM 21582*	72.79	82.52	16.5	18.1	16.3
*D. fermentans DSM 18053*	*D. beijingensis DSM 21582*	81.41	85.13	40.4	25.3	35.5

**Figure 3:**
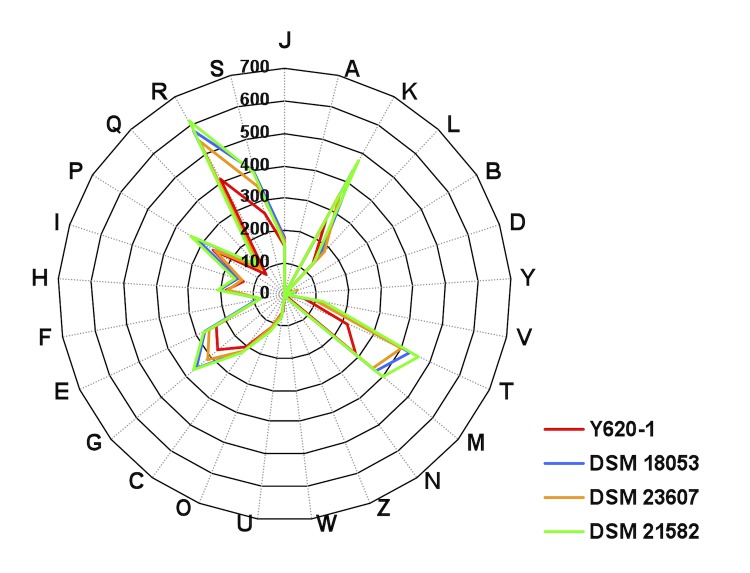
Distribution of COG functional categories in the genome of *Dyadobacter tibetensis* (T) Y620-1, *D. alkalitolerans* DSM 23607, *D. fermentans* DSM 18053 and *D. beijingensis* DSM 21582.

ANI analysis showed that strain Y620-1 shared a low degree of similarity with other *Dyadobacter* species (< 69% ANIb and < 84% ANIm), whereas relatively higher ANI value were obtained for *D. alkalitolerans* DSM 23607, *D. fermentans* DSM 18053 and *D. beijingensis* DSM 21582 (Table 5). Although the core concept of GGDC was based on ‘genome blast distance phylogeny’, which is different from ANI [35], GGDC analysis showed similar results. In both analyses, the highest similarity values were observed in the comparisons of *D. fermentans* DSM 18053 with *D. beijingensis* DSM 21582. These results were in line with phylogeny analysis based on 16S rRNA gene, which shows that *D. fermentans* DSM 18053 and *D. beijingensis* DSM 21582 form a cluster with *Dyadobacter soli* MJ20. Moreover, the comparison of distribution of COG categories in the genome of four *Dyadobacter* strains revealed that there were significant correlations between the distribution of COG categories of strain Y620-1 and other strains (r = 0.970-0.979). However, relatively higher correlation coefficients were observed for *D. alkalitolerans* DSM 23607 and *D. fermentans* DSM 18053 (0.988), *D. alkalitolerans* DSM 23607 and *D. beijingensis* DSM 21582 (0.991), and *D. fermentans* DSM 18053 and *D. beijingensis* DSM 21582 (0.991) (Figure 3).

Five cold-shock proteins were found in this genome including *CspA*, *GyrA*, *RbfA*, and *NusA*. The proteins coded by gene *RecA*, *RecF*, *RecG*, *RecN*, *RecO*, *RecQ*, *RadA* and *RadC*, which play a critical role in recombinational repair of damaged DNA, were also found [36]. Single-stranded-DNA-specific exonuclease RecJ, required for many types of recombination events [37], and CRISPRs Cas1, interacts with components of the DNA repair systems, also were found [38]. Phage shock protein C existed in the genome, which may play a significant role in the competition for survival under nutrient- or energy-limited conditions [39]. When bacteria deposit on the glacier, the low temperature, high UV radiation, and desiccation could induce the cold-shock and recombinational repair of damaged DNA proteins. Additionally, oligotrophic condition of glacial ice may induce the phage shock protein C. The genome sequence of strain Y620-1 provides genetic information to identify the genes linked to its specific mechanisms for adaption to extreme glacial environment.
